# Growth and Biochemical Composition Characteristics of *Arthrospira platensis* Induced by Simultaneous Nitrogen Deficiency and Seawater-Supplemented Medium in an Outdoor Raceway Pond in Winter

**DOI:** 10.3390/foods10122974

**Published:** 2021-12-03

**Authors:** Hualian Wu, Tao Li, Jinting Lv, Zishuo Chen, Jiayi Wu, Na Wang, Houbo Wu, Wenzhou Xiang

**Affiliations:** 1 CAS Key Laboratory of Tropical Marine Bio-Resources and Ecology, Guangdong Key Laboratory of Marine Materia Medica, Institution of South China Sea Ecology and Environmental Engineering, South China Sea Institute of Oceanology, Chinese Academy of Sciences, Guangzhou 510301, China; hlwu@scsio.ac.cn (H.W.); taoli@scsio.ac.cn (T.L.); lv_jt2017@126.com (J.L.); 18390943716@163.com (Z.C.); kayeewu@scsio.an.cn (J.W.); nawang@scsio.ac.cn (N.W.); wuhoubo@scsio.ac.cn (H.W.); 2 Southern Marine Science and Engineering Guangdong Laboratory (Guangzhou), Guangzhou 511458, China

**Keywords:** *Arthrospira* *platensis*, nitrogen deficiency, biochemical composition, areal productivity, carbohydrate content, protein content

## Abstract

*Arthrospira platensis*, a well-known cyanobacterium, is widely applied not only in human and animal nutrition but also in cosmetics for its high amounts of active products. The biochemical composition plays a key role in the application performance of the *Arthrospira* biomass. The present study aimed to evaluate the growth and biochemical composition characteristics of *A. platensis*, cultured with a nitrogen-free and seawater-supplemented medium in an outdoor raceway pond in winter. The results showed that the biomass yield could achieve 222.42 g m^−2^, and the carbohydrate content increased by 247% at the end of the culture period (26 d), compared with that of the starter culture. The daily and annual areal productivities were 3.96 g m^−2^ d^−1^ and 14.44 ton ha^−1^ yr^−1^ for biomass and 2.88 g m^−2^ d^−1^ and 10.53 ton ha^−1^ yr^−1^ for carbohydrates, respectively. On the contrary, a profound reduction was observed in protein, lipid, and pigment contents. Glucose, the main monosaccharide in the *A. platensis* biomass, increased from 77.81% to 93.75% of total monosaccharides. Based on these results, large-scale production of carbohydrate-rich *A. platensis* biomass was achieved via a low-cost culture, involving simultaneous nitrogen deficiency and supplementary seawater in winter.

## 1. Introduction

*Arthrospira*, also known as *Spirulina*, is a multicellular and filamentous cyanobacterium that can adapt to different water environments, such as freshwater, brackish lakes, and alkaline saline lakes [1]. *Arthrospira* has drawn more and more attention in food supplements, animal feed additives, pharmaceutical products, and cosmetics, due to its high levels of natural products with nutritional and health benefits, such as protein, phycocyanin, polysaccharides, polyunsaturated fatty acids (PUFAs), pigments, and amino acids [2,3,4]. *Arthrospira* has become one of the most important cultured microalgal species globally, with a commercial production of 89,000 tons in 2016 [5].

*Arthrospira* is rich in protein (up to 60% DW, dry weight), polysaccharides (15–25% DW), lipids/PUFAs (3–9% DW), and pigments (mainly phycocyanin and total carotenoids) [6]. Proteins from *Arthrospira* have balanced essential amino acids and can be hydrolyzed to bioactive peptides associated with healthy food, pharmaceuticals, and nutraceuticals [7]. *Arthrospira* polysaccharides with functional properties have been applied to cosmeceutical, nutraceutical, pharmaceutical industries, etc. [8,9]. Carbohydrates, the photosynthetic products of *Arthrospira*, can be used as a renewable source for producing bioethanol [10]. Phycocyanin, being a special pigment protein, has been used not only as a natural colorant in various sweets and drinks but also in food/pharmaceutical products and fluorescent markers for its positive health effects and fluorescence spectra characteristics, respectively [11,12]. Consequently, the biochemical composition plays a key role in the application directions of the *Arthrospira* biomass. However, the growth and biochemical composition of *Arthrospira* were influenced by many factors, such as nutrients, temperature, light, and salinity [13,14,15]. Strategies for regulating these factors have been developed to increase the accumulation of high-value products in *Arthrospira* for different application purposes [16,17]. However, little information is known about the combined effects of different environmental factors on the biochemical compositions of *Arthrospira* cultured in large-scale outdoor raceway ponds.

Lowering the *Arthrospira* production costs is a goal that manufacturers and researchers want to achieve. The culture medium is one of the main costs of *Arthrospira* biomass production, accounting for about 35% of the total cost [18]. Besides, supplementing seawater also contains trace elements, such as manganese, phosphorus, potassium, iron, zinc, boron, sulfur, chlorine, and calcium [17], which are essential nutrients for the growth of microalgae. Previous studies showed that *Arthrospira* could utilize seawater as a nutrient source [17,19]. Clearly, partially substituting freshwater with seawater could lower fertilizer costs and reduce microbial contaminants and heavy metal pollutants [19]. Therefore, the use of a seawater-supplemented medium for reducing the production cost of *Arthrospira* is a feasible strategy. On the other hand, changes of salinity have been shown to induce the characteristics of the nutritional properties in microalgae. The biologically-active compounds in *Arthrospira* were induced by salt stress conditions [20]. In addition, a medium using seawater reduces the dependence of *Arthrospira* on freshwater, which promotes the development of *Arthrospira* culture in coastal shoals or where freshwater is scarce.

One of the most striking features of *Arthrospira* is its rapid growth under suitable conditions. Furthermore, *Arthrospira* shows strong adaptability to relative extreme environments and effectively avoids contaminations from other microalgae, bacteria, and protozoa. A raceway paddle wheel mixed pond is a cost-effective method that has been used in the commercial production of *Arthrospira* on a large scale [21]. However, the main disadvantage of open raceway ponds is that open systems are particularly dependent on the weather/climate. *Arthrospira* is widely cultured in tropical and subtropical areas in the world, since its species shows an optimal growth temperature in the range of 35–37 °C [22]. China is the largest producer of *Arthrospira*, holding about two-thirds of worldwide *Arthrospira* production, and its production is still growing [21]. However, the producers of *Arthrospira* biomass in China are distributed in the Hainan, Guangxi, Fujian, Zhejiang, Yunnan, Jiangsu, Jiangxi, and Inner Mongolia provinces, throughout tropical, subtropical, and temperate areas [21]. The commercial production of *Arthrospira* in subtropical and temperate areas of China is affected by seasonality. Consequently, *Arthrospira* is only cultured all year round in the Hainan, Guangdong, and Guangxi provinces. Even cultured in the tropics, *Arthrospira* is also subjected to unsuitable environmental conditions in winter, such as low temperature and insufficient light intensity, which contribute to a negative impact on the yield and quality of *Arthrospira* biomass. Nitrogen is a primary nutrient for algal growth. Reducing nitrate use, as much as possible, is beneficial for improving the cultivation cost of *Arthrospira*. However, nitrogen limitations can increase the accumulation of lipids or carbohydrates [16,23] but lower protein and chlorophyll content in *Chlorella vulgaris* L3 [24]. A study has shown that the growth and biochemical compositions of the brown alga *Sargassum fusiforme* were significantly affected by the interactions between temperature and salinity [25]. However, there is little information on the combined effects of nitrogen deficiency and seawater supplementation on the production of *Arthrospira* in large-scale raceway ponds systems in winter with unsatisfactory weather. 

In the present study, freshwater *A. platensis* was cultured in an outdoor raceway pond under combined stresses from nitrogen deficiency, seawater supplementation, and a winter climate with unsuitable temperatures. The production performance of biomass and biochemical and nutritional composition of this microalga, in response to such conditions, was evaluated. This work not only provides information for the large-scale production of *Arthrospira* in winter, but also provides a strategy for improving carbohydrate accumulation in a low-cost and simple manner.

## 2. Materials and Methods

### 2.1. Algal Strain and Culture Conditions

**Figure 1:**
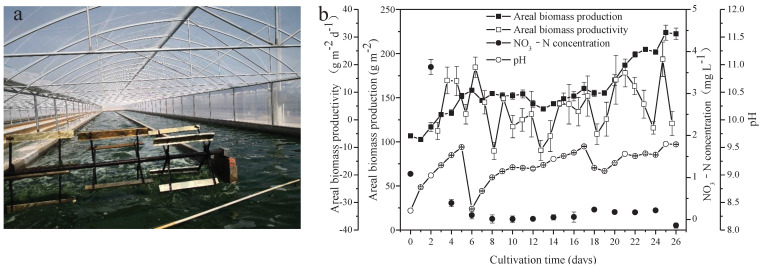
Scene picture of *A. platensis* culture (**a**) and growth characteristics of *A. platensis*, as a function of culture time (**b**) (*n* = 3; data: mean ± standard deviations).

*A. platensis*, cultured for commercial production in a *Arthrospira* cultivation company (Zhongshan Cyanobacteria Biological Food Development Co., Ltd Beihai Branch, Beihai, China), was used as the microalgal seed for this experiment in the same company. The microalgae were cultured in an outdoor raceway pond, with dimensions of 95 m in length, 8 m in width, and 0.15 m in depth, and a working volume of approximately 120 m^3^ (Figure 1a). Paddle wheels were used to circulate and mix the cultures at a speed of 16 revolutions per minute. The microalgal seed was harvested and washed with groundwater and then inoculated into nitrogen-free medium, which was supplemented with seawater (salinity of 35‰) in volume ratios of 1:7, in order to obtain an initial cell biomass of approximately 0.59 g L^−1^. The medium consisted of (m^−3^): NaHCO_3_, 5 kg; FeSO_4_·7H_2_O, 0.01 kg; and H_3_PO_4_, 0.1 kg. The cultures were subjected to natural sunlight and temperature from 29 December 2020 to 24 January 2021. The light intensity and temperature of this culture system were recorded daily (at 8:00, 14:00, and 17:00) using a temperature gauge and TES-1332A digital luminance meter, respectively. Data are shown in Figure S1. During the cultivation process, the salinity of the medium was maintained between 9‰ and 12‰ by supplementing groundwater, while the pH was controlled in a range of 8.3 to 9.5, most of the time above 9, by bubbling 100% CO_2_ into the medium.

### 2.2. Characterization of the Water Sample

Elements in seawater including iron (Fe), sodium (Na), potassium (K), calcium (Ca), magnesium (Mg), lead (Pb), cadmium (Cd), arsenic (As), and phosphorus (P) were detected with an optima 2000DV inductively coupled plasma–atomic emission spectrometer (PerkinElmer Inc., Waltham, MA, USA). Copper (Cu), zinc (Zn), mercury (Hg), and chromium (Cr) were tested, in accordance with the GB 17378.4–2007 standard. All procedures were in accordance with the specification for the oceanographic survey of China. The detection limit was 0.00005–0.050 mg L^−1^.

During the culture period, culture collected every two days was filtered through a 0.22 μM filter membrane. The NO_3_^−^ concentration in the filtrate was measured by the ultraviolet spectrophotometric method [26]. The absorbances of the filtrate at 220 nm and 275 nm were recorded using a TU-1810 spectrophotometer (Persee, Beijing, China). The NO_3_^−^ absorbance values were calculated using the equation: A = A_220_ − 2 × A_275_ and converted to NO_3_^−^ concentrations using the calibration curve, obtained by the NO_3_^−^ standards.

### 2.3. Growth Measurements

Algal biomass, shown as dry weight (DW), was determined daily. DW was measured by filtering a 20-mL sample through a pre-weighed filter membrane. The filters were dried in an XMTD-8222 drying oven (Jinghong, Shanghai, China) at 80 °C until a constant weight was achieved. Furthermore, the key indexes of microalgal productivity were also evaluated by the following formulas:

Volumetric biomass productivity = (B_t26_ − B_t0_)/T

Areal biomass productivity = (B_t26_ − B_t0_) × D/T

Volumetric carbohydrate productivity = (B_t26_ × C_t26_ − B_t0_ × C_t0_)/T

Areal carbohydrate productivity = (B_t26_× C_t26_ − B_t0_ × C_t0_)/T

Volumetric protein productivity = (B_t26_ × P_t26_ − B_t0_ × P_t0_)/T

Areal protein productivity = (B_t26_ × P_t26_ − B_t0_ × P_t0_) × D/T

Volumetric lipid productivity = (B_t26_ × L_t26_ − B_t0_ × L_t0_)/T

Areal lipid productivity = (B_t26_ × L_t26_ − B_t0_ × L_t0_) × D/T

Volumetric phycobiliprotein productivity = (B_t26_ × PB_t26_ − B_t0_ × PB_t0_)/T

Areal phycobiliprotein productivity = (B_t26_ × PB_t26_ − B_t0_ × PB_t0_) × D/T

wherein B_t26_ and B_t0_ were the biomass concentrations (g L^−1^) on day 26 and day 0, respectively. C_t26_ and C_t0_ were the contents of carbohydrates on day 26 and day 0, respectively. P_t26_ and P_t0_ were the contents of protein on day 26 and day 0, respectively. L_t26_ and L_t0_ were the contents of lipid on day 26 and day 0, respectively. PB_t26_ and PB_t0_ were the contents of phycobiliprotein on day 26 and day 0, respectively. T was the overall culturing time (26 d), while D represented the culturing depth of the raceway pond in meters (0.15 m).

### 2.4. Microalgal Biochemical Composition

Protein content, carbohydrate content, lipid content, pigment content, fatty acid composition, amino acid composition, and monosaccharide composition in the microalgal biomass were tracked during the culture period. Microalgal wet biomass was collected through a 400 mesh sieve, rinsed with groundwater, and then freeze-dried by an FD-1-50 freeze dryer (Boyikang, Beijing, China). The biomass powder was kept at −20 °C for biochemical analysis.

#### 2.4.1. Carbohydrate Content

The *A. platensis* biomass (10 mg) was extracted by acid hydrolysis, with 0.5 N H_2_SO_4_ at 80 °C for 2 h, and this process was repeated three times. The carbohydrates content was measured by the phenol-sulfuric acid method [27].

#### 2.4.2. Total Lipid Content

The *A. platensis* biomass (200 mg) was used to determine total lipid content. Total lipids in the *A. platensis* biomass were determined by gravimetric methods after extraction with dimethyl sulfoxide-methanol (1:9, *v/v*) and hexane-diethyl ether (1:1, *v/v*), according to the method described by Khozin-Goldberg et al. [28].

#### 2.4.3. Protein Content

Proteins in the free lipid residue were extracted with 0.5 N NaOH at 80 °C for 30 min, and the process was repeated four times. Protein content was estimated colorimetrically using the Lowry method [29], and a protein quantitation kit was offered by Nanjing Jiancheng Biological Engineering Institute.

#### 2.4.4. Ash Content

Microalgal freeze-dried biomass (1 g) was combusted at 650 °C for 2 h in an SX2-4-13 muffle furnace (Baixin, Fushun, China). Then, the ash was accurately determined by gravimetric method.

### 2.5. Assessment of Pigments

Fat-soluble pigments in *A. platensis* were extracted with 100% methanol. The absorbance of chlorophyll *a* and total carotenoids in the supernatant was measured at 665.2 nm, 652.4 nm, and 470 nm using a TU-1810 spectrophotometry (Persee, Beijing, China); then, the concentrations were calculated using the method previously described by Li et al. [30].

The phycobiliproteins, including phycocyanin (PC) and allophycocyanin (APC), in *A. platensis* were extracted with a 0.1 M phosphate buffer (pH 6.7) by a freeze–thawing method, until the supernatant had no blue color. The absorbance of the supernatant was measured at 615 nm and 652 nm using a TU-1810 spectrophotometer (Persee, Beijing, China). The concentrations of PC and APC were calculated according to Bennett and Bogorad [31]. The amount of individual pigments was shown as mg g^−1^ dry matter.

### 2.6. Evaluation of Fatty Acid Composition

For fatty acid (FA) analysis, the *A. platensis* biomass was transmethylated with 2% H_2_SO_4_ in a methanol/toluene mixture (90:10, *v/v*) at 80 °C for 1.5 h. Heptadecanoic acid (C17:0) was added as an internal standard. Then, an analysis of fatty acid methyl esters (FAMEs) was carried out using a Shimadzu-2014C gas chromatograph, equipped with a flame ionization detector (Shimadzu, Kyoto, Japan) and fused silica capillary column DB-WAX (30 m × 0.25 mm). The injection port temperature was 260 °C. The column temperature was programmed from 190 °C (with a hold of 5 min) to 250 °C (with a hold of 5 min), at a rate of 5 °C min^−1^. The amount of individual FAMEs was calculated according to the method described by Li et al. [32].

### 2.7. Estimation of Amino Acid Composition

The amino acid composition of the *A.* *platensis* biomass was determined according to the Chinese national standard method (Chinese Standard GB 5009.124–2016 and GB/T 18246-2000). The amino acid analysis was performed using a LA8080 amino acid analyzer (Hitachi, Tokyo, Japan) after acid hydrolysis with 6 M HCl at 110 °C for 22 h, except for tryptophan and cysteine. Amounts of tryptophan and cysteine were analyzed by Agilent-1260 high-performance liquid chromatography (Agilent Technologies, Santa Clara, CA, USA) after alkaline hydrolysis with 4 M LiOH at 110 °C for 20 h. The content of individual amino acids was quantified from the peak area ratio of individual amino acids and external standards. The amino acid composition was shown as amino acid g/100 g dry matter.

### 2.8. Determination of Monosaccharide Composition

For the monosaccharide composition, freeze-dried microalgal biomass was hydrolyzed with 3 M trifluoroacetic acid at 120 °C for 3 h. Then, hydrolysis solution was dried by nitrogen blow. After these processes of dissolution, dilution, and centrifugation, the hydrolyzed product in the supernatant was analyzed directly by ICS5000 Thermo Fisher IC system (Thermo Fisher, Waltham, MA, USA), equipped with a DionexTM CarbopacTM PA20 column (150 mm × 3 mm) and an electrochemical detector. The temperature of the column was 30 °C. A (deionized water), B (NaOH, 0.015 M), and C (NaOH, 0.015 M; NaOAC, 0.1 M) were used as the mobile phases, with gradient elution at a flow rate of 0.3 mL min^−1^. The gradient was as follows: 0–18 min (98.8% A and 1.2% B), 20–30 min (50% A and 50% B), 30.1–46 min (100% C), 46.1–50 min (100% B), and 50.1–80 min (98.8% A and 1.2% B). The injection volume was 5.0 μL. Standard monosaccharides, including rhamnose (Rha), glucosamine (GlcN), galactose (Gal), glucose (Glc), glucuronic acid (GlcA), and xylose (Xyl), were analyzed under the same conditions, as a reference.

### 2.9. Statistical Analysis

All data in the study are presented as means values ± standard deviations. Statistical analysis (ANOVA) was performed using SPSS software version 20.0 (SPSS, Chicago, IL, USA), in order to confirm inter-group variation.

## 3. Results

### 3.1. Growth Characteristics of A. platensis

Seawater used in the present study was rich in Na (8531.667 mg L^−1^), K (303.318 mg L^−1^), Mg (824.333 mg L^−1^), and Ca (240.399 mg L^−1^). Furthermore, other important elements necessary for metabolism in microalgae, including Fe (0.008 mg L^−1^) and P (0.029 mg L^−1^), were found in the seawater. Heavy metal elements, including Pb, Cd, As, Hg, and Cr, were not detected (Table 1). Freshwater *A. platensis* was successfully cultured in the medium with the nutrients supplemented by NaHCO_3_, FeSO_4_, H_3_PO_4_, and seawater in a raceway pond (Figure 1a). As shown in Figure 1b, the areal production of *A. platensis*, shown as the biomass concentration, increased with culture time from 106.68 g m^−2^ to 222.42 g m^−2^ after 26 days. The biomass productivity varied from −11.33 g m^−2^ d^−1^ to 21.97 g m^−2^ d^−1^ during the whole cultivation process. The concentrations of nitrate-nitrogen were always extremely low throughout the cultivation. Due to cellular metabolisms, to absorb CO_2_ from the medium, pH in the culture medium steadily rose higher. In this study, CO_2_ was supplied to the *A. platensis* culture, in order to adjust the pH values between 8.3 and 9.5—most of the time, above 9 (Figure 1b).

### 3.2. Biochemical Composition and Yield of A. platensis

**Figure 2:**
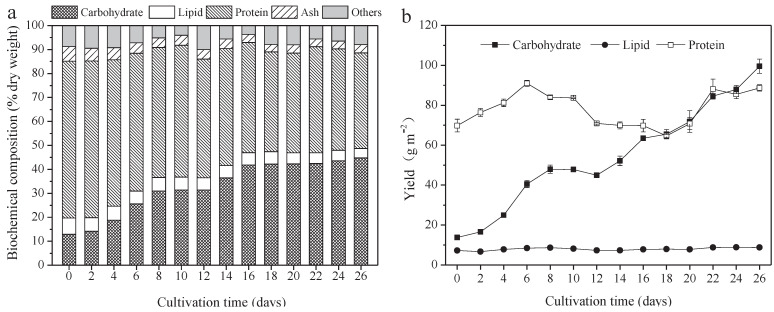
Biochemical composition and yield of *A. platensis*, as a function of culture time. (**a**) Biochemical composition and (**b**) yield (*n* = 3; data: mean ± standard deviations; DW: dry weight).

The protein, carbohydrate, lipid, and ash contents of *A. platensis*, cultured in the raceway pond and determined every 2 days, are shown in Figure 2a. In the *A. platensis* biomass, proteins were the most-reduced substances, followed by lipid and ash. During the culture period, the content of proteins, lipids, and ash in *A. platensis* decreased significantly (*p* ≤ 0.01), from 65.4% DW to 39.9% DW, 6.8% to 4.0% DW, and 6.1% to 3.4% DW, respectively. On the contrary, the carbohydrate content was 12.9% DW on day 0 and rapidly increased to 42.2% DW on day 18 (*p* ≤ 0.01); thereafter, no significant changes were observed (*p* > 0.05). Figure 2b offers information about the changes in carbohydrate, protein, and lipid yields. The yields of all the main three constituents showed an increasing tendency as the culture time increased. The carbohydrate yield was the highest, followed by protein and lipid. Carbohydrate yield achieved a maximum of 99.6 g m^−2^ on day 26, 7.2 times that of day 0. Protein yield, however, increased from 69.8 g m^−2^ to 88.7 g m^−2^, only showing an 18.9% improvement. In contrast, lipid yield was far less than the carbohydrate and protein yield, only 8.8 g m^−2^ on day 26.

### 3.3. Changes in Fatty Acids Composition

**Figure 3:**
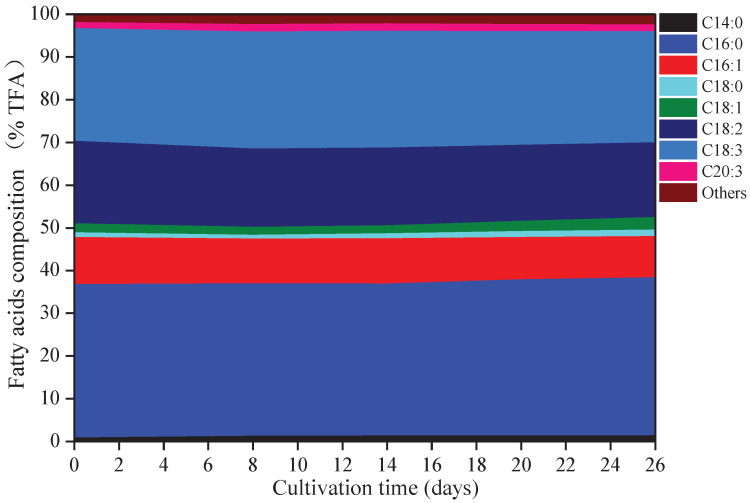
Fatty acids composition of *A. platensis*, as a function of culture time (*n* = 3; data: mean; TFA: total fatty acids).

Eight kinds of fatty acids were observed in *A. platensis,* including myristic acid (C14:0), palmitic acid (C16:0), palmitoleic acid (C16:1), octadecanoic (C18:0), oleic acid (C18:1), linoleic acid (C18:2), γ-linolenic acid (C18:3), and eicosatrienoic acid (C20:3) (Figure 3). C16:0 was the primary fatty acid in *A. platensis*, comprising of 35.6% to 37.0% of the TFA (total fatty acids), followed by C18:3, C18:2, and C16:1 at the ranges of 26.0–27.4% TFA, 17.5–19.3% TFA, and 9.7–11.0% TFA, respectively. Among these fatty acids, few changes were observed in the proportions of C16:0 and C18:3, while the proportions of C14:0, C16:1, C18:0, C18:1, C18:2, and C20:3 changed. Such culture conditions promoted the accumulation of C14:0, C18:0, C18:1, and C20:3, with increases of 70.86%, 36.41%, 39.35%, and 17.12% over that of day 0 (*p* ≤ 0.05), respectively.

### 3.4. Variations of Pigment Content

**Figure 4:**
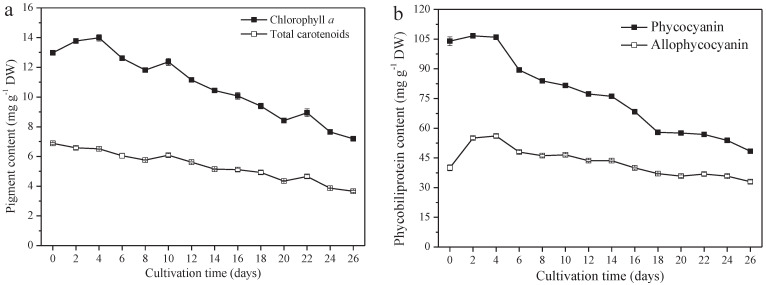
Pigment content of *A. platensis*, as a function of culture time. (**a**) Fat−soluble pigments and (**b**) phycobiliprotein (*n* = 3; data: mean ± standard deviations).

*A. platensis* produces high-value pigments, including PC, APC, chlorophyll *a*, and carotenoids. PC and APC are water-soluble protein pigments. Chlorophyll *a* and carotenoids are fat-soluble pigments. The contents of these four pigments all decreased significantly (*p* ≤ 0.01). From day 0 to day 26, the PC content decreased from 104.0 mg g^−1^ DW (day 0) to 48.4 mg g^−1^ DW (day 26), 40.0 mg g^−1^ DW to 33.0 mg g^−1^ DW, 13.0 mg g^−1^ DW to 7.2 mg g^−1^ DW, and 6.9 mg g^−1^ DW (day 0) to 3.7 mg g^−1^ DW for APC chlorophyll *a* and the carotenoids, respectively (Figure 4a,b). The variation of PC content among them was the greatest; a 53.5% reduction was observed on day 26, followed by carotenoids, chlorophyll *a*, and APC, at a reduction of 46.4%, 44.6%, and 17.5%, respectively.

### 3.5. Alterations of Amino Acids Composition

Amino acids composition in *A. platensis* (g/100 g dry matter) is shown in Table 2. Five amino acids were mainly present in *A. platensis,* including glutamic acid, aspartic acid, leucine, alanine, and arginine. The quality of proteins was evaluated by essential amino acids. *Arthrospira* is a complete protein source, containing all eight essential amino acids, which significantly decreased during the culture period (*p* ≤ 0.05). The results, regarding essential and nonessential amino acids in the *A. platensis* biomass, are summarized in Table 2. For essential amino acids, leucine had a large proportion, followed by valine, with a proportion of 5.80 and 4.04 g/100 g dry matter, respectively, and then declined to 2.93 and 2.12 g/100 g dry matter at the end of the cultivation process, respectively. The results for nonessential amino acids in *A. platensis* at different stages of cultivation indicated glutamic acid, aspartic acid, alanine, and arginine were substantial amounts, followed by glycine and serine. The ratio coefficient scores of amino acids (SRC) in *A. platensis*, calculated according to the FAO/WHO standard, were 49.94, 47.73, and 46.22 for days 0, 14, and 26, respectively.

### 3.6. Changes of Monosaccharide Composition

The monosaccharide composition in the *A. platensis* biomass was determined after full hydrolysis of the biomass by ion chromatography. The results showed six monosaccharides in the *A. platensis* biomass, including Glc, Rha, GlcN, Gal, Xyl, and GlcA. Carbohydrates in the *A. platensis* biomass were predominantly composed of Glc, which drastically increased from 77.81% to 93.75% during the culture period (*p* ≤ 0.05) (Table 3). The percentage of Glc and Xyl increased gradually with the increase in culture time, while the other four monosaccharides decreased.

### 3.7. Biomass and Active Substances Productivity of A. platensis

The production performance of *A. platensis* cultured in nitrogen-free and seawater-supplemented medium in winter was assessed by a combined evaluation of the productivity of biomass, carbohydrates, proteins, lipids, and phycobiliproteins. In the present study, the volumetric productivity, areal productivity, and annual productivity of the *A. platensis* biomass were 24.57 mg L^−1^ d^−1^, 3.96 g m^−2^ d^−1^, and 14.44 ton ha^−1^ yr^−1^, respectively (Table 4). In terms of the substances in biomass, the volumetric and areal productivity of carbohydrates were the highest, at 17.92 mg L^−1^ d^−1^, and 2.88 g m^−2^ d^−1^, respectively, followed by the proteins, phycobiliproteins, and lipids (Table 4).

## 4. Discussion

*Arthrospira* is a cyanobacterium that has been commercialized worldwide for health foods and therapeutic purposes, due to its valuable substances, such as proteins and polysaccharides [33]. As the awareness of the importance of natural products is growing, high-value substances from *Arthrospira* are used precisely in different fields, according to their characteristics. Therefore, the biochemical compositions of *Arthrospira* directly affect the application direction of this microalgal biomass in the downstream industry chain. For example, the biomass with high phycocyanin content can be specially used for the development of natural food colors or other phycocyanin products, while the one containing high carbohydrates content has potential as a source for biofuel generation or active polysaccharides production. However, even though it was associated with genetic characteristics of algal strains, the substances contained in the *Arthrospira* biomass were influenced by factors such as amounts of nutrients (fertilizers), temperature, and light intensity [13,15,20,34,35].

In the present study, *A. platensis* was subjected to a nitrogen-free and seawater-supplemented medium. The seawater not only provides some important elements for the metabolism, such as magnesium and some trace elements, but also increases the salinity of the medium. Freshwater *A. platensis* was successfully cultured in such a medium mentioned above, which means *Arthrospira* is highly adaptable to salinity; our finding was consistent with Jiang et al. [36]. Nitrogen limitation and high salinity have been reported to have negative effects on the growth of *A. platensis* [35,37]. In contrast, in our study, biomass production showed a general increase during the culture period, increasing by 108%, compared to the initial level, reaching 222.42 g m^−2^. The net increase in the biomass concentration was 115.74 g m^−2^. In terms of the areal biomass productivity, the fluctuation changed sharply. The maximum biomass productivity occurred on day 25, with 21.97 g m^−2^ d^−1^, but the average biomass productivity (3.96 g m^−2^ d^−1^) was much lower than previously reported [16,38]. The medium components and environmental factors largely affect the growth [13,15,20,34,35]. The growth, shown as biomass production and productivity in our study, was significantly lower than the results from those studies mentioned above, which was mainly attributed to the following reasons. Firstly, long-term nitrogen stress, caused by nitrogen deficiency and salt stress, partly inhibited the growth results in the low biomass productivity of *A.platensis*. Secondly, neither the solar radiation nor sunshine time in winter was optimal for *A.platensis* growth. Additionally, our experiment was conducted in winter; thus, the temperature was far below the optimum temperature for culturing *A*. *platensis*.

The environmental conditions have a major impact on the biochemical composition of microalgae [23]. Under such cultured conditions, in the present study, the contents of the protein, lipid, and photosynthetic pigments, including PC, APC, chlorophyll *a*, and carotenoids, in *A*. *platensis* decreased with the prolonging of culture time. On the contrary, the carbohydrate content accumulated up to 44.78% DW, far higher than the normal content of carbohydrates in *Arthrospira* (12–20% DW) [1]. Nitrogen/phosphorus nutrient limitation, as well as salinity stress, have been reported to cause an increase in the carbohydrate content, and, inversely, a decrease in protein in *Arthrospira* [39,40,41,42,43]. However, even though the *A. platensis* were all cultured in industrial-scale raceway ponds, the carbohydrate content value in this study was lower than that reported by Liu et al. [16]. It was possible that, in the present study, lower temperatures and less light intensity in winter, which the *A*. *platensis* were subjected to, imposed a negative impact on the accumulation of high carbohydrate content. Therefore, in this study, nitrogen deficiency, high salinity from seawater, and unfavorable climate, acting synergistically, resulted in an increase in the carbohydrates and decrease in the protein and lipid content.

The main five amino acids present in *A. platensis* included glutamic acid, aspartic acid, leucine, alanine, and arginine, which was consistent with a previous study reported by Bashir et al. [44]. However, Uslu et al. reported that the five predominant amino acids of *A. platensis*, in previous studies, were glutamic acid, aspartic acid, leucine, valine, and isoleucine [45]. The main reason for the discrepancy of these results is mostly attributable to differences in the culture condition and genetic characteristics of the algal strains used in these studies. *Arthrospira* is a complete protein source, containing all these essential amino acids [2]. Our results revealed that the combined effects of nitrogen deficiency, high salinity from seawater, and unfavorable climate would lead to a decreased content of the 18 kinds of amino acids in *Arthrospira,* including the essential and nonessential amino acids. The content of individual amino acids decreased as culture time increased. It was reported that amino acids were degraded under nitrogen stress in diatom *Phaeodactylum tricornutum* [46]. This biochemical process could promote the redistribution of carbon and nitrogen flow [39] and improve triacylglycerol biosynthesis in *P*. *tricornutum* [47] and *Chlamydomonas reinhardtii* [48]. The decrease in amino acids composition in *A*. *platensis* was accompanied by the accumulation of carbohydrates, which means the redistribution of carbon and nitrogen flow under such culture conditions.

Six kinds of monosaccharides, including Glc, Rha, GlcN, Gal, Xyl, and GlcA, were present in the *A. platensis* biomass. During the culture period, Glc was the dominant monosaccharide (77.81–93.75%). The major carbohydrates are polysaccharides in *Arthrospira* [49]. Glc, as the main component of the polysaccharides of *A. platensis*, was consistent with a previous study [16]. Environmental factors, such as irradiance, temperature, nitrate, and phosphate, affected the monosaccharide composition; for example, low nitrate concentration and high culture temperature increased the monosaccharide contents. Our study results showed that the combined stress of nitrogen deficiency, salinity stress, and adverse weather contributed to the ratio changing of monosaccharide composition. Under the culture conditions in the present study, the main carbohydrate in *A. platensis* was probably polysaccharides, with Glc being the main monosaccharide.

The volumetric and areal biomass productivities of *A. platensis* (24.57 mg L^−1^ d^−1^ and 3.96 g m^−2^ d^−1^, respectively) in our study were lower than that of a previous study (100.8 mg L^−1^ d^−1^ and 27.5 g m^−2^ d^−1^, respectively) [16]. The average annual productivity (14.44 tons ha^−1^ yr^−1^) in our study compares very unfavorably with the reports for cultivation with nutrients from wastewater during summer (39.8 tons ha^−1^ yr^−1^), but the maximum annual productivity (80.19 tons ha^−1^ yr^−1^) is higher than that of previous report (51.52 tons ha^−1^ yr^−1^) [50]. The culture conditions detailed in the present study resulted in low lipid, protein, and phycobiliprotein content, as well as productivity; thus, they are not suitable for the production of the *A. platensis* biomass for PUFAs, protein resources, and phycocyanin products. Regarding carbohydrates in *A. platensis*, the carbohydrate content and volumetric and areal productivity were 44.78%, 17.92 mg L^−1^ d^−1^, and 2.88 g m^−2^ d^−1^, respectively; the annual productivity was 10.53 ton ha^−1^ yr^−1^. Consequently, the *A. platensis* biomass showed potential as a carbohydrate source for active polysaccharides and biofuel generation.

## 5. Conclusions

*A. platensis* can be cultivated with a nitrogen-free and seawater-supplemented medium in an outdoor raceway pond with low temperatures. The biochemical composition, namely, the main natural nutrients in *A. platensis,* were significantly affected by the combined stress of nitrogen deficiency, salinity stress, and adverse weather. The carbohydrate-enriched biomass of *A. platensis* was obtained at an industrial scale by this low-cost and simple culture method. The carbohydrate-enriched biomass can be used to develop novel functional foods with biologically-active polysaccharides or other purposes, such as bioethanol production and cosmetic applications.

## Figures and Tables

**Table 1:** Chemical elements in the seawater used in the present study.

Element	Concentration (mg L^−1^)	Detection Limit (mg L^−1^)
Fe	0.008 ± 0.000	0.001
Na	8531.667 ± 239.201	0.001
K	303.318 ± 7.946	0.001
Ca	240.399 ± 7.809	0.001
Mg	824.333 ± 37.554	0.001
P	0.029 ± 0.016	0.010
Pb	ND	0.001
Cd	ND	0.001
As	ND	0.006
Cu	ND	0.005
Zin	ND	0.010
Hg	ND	0.00005
Cr	ND	0.050

Fe: iron, Na: sodium, K: potassium, Ca: calcium, Mg: magnesium, Pb: lead, Cd: cadmium, As: arsenic, Cu: Copper, Zn: zinc, Hg: mercury, Cr: chromium, ND: not detected.

**Table 2:** Amino acids composition and the ratio coefficient score of *A. platensis* during culture time.

Amino Acid Type	Amino Acid Content (g/100 g Dry Matter)		
	Day 0	Day 14	Day 26
Essential amino acids	25.85 ± 1.15	18.14 ± 0.06	12.99 ± 0.37
Phenylalanine	2.84 ± 0.12	2.06 ± 0.01	1.45 ± 0.03
Methionine	1.22 ± 0.04	0.69 ± 0.01	0.43 ± 0.00
Threonine	3.27 ± 0.14	2.29 ± 0.04	1.65 ± 0.06
Valine	4.04 ± 0.19	2.86 ± 0.00	2.12 ± 0.05
Isoleucine	3.53 ± 0.16	2.47 ± 0.01	1.75 ± 0.06
Leucine *	5.80 ± 0.26	4.08 ± 0.01	2.93 ± 0.09
Lysine	3.350 ± 0.170	2.395 ± 0.021	1.700 ± 0.057
Histidine	0.98 ± 0.06	0.70 ± 0.00	0.50 ± 0.02
Tryptophan	0.835 ± 0.021	0.630 ± 0.014	0.475 ± 0.007
Nonessential amino acids	36.43 ± 1.54	25.17 ± 0.30	17.98 ± 0.43
Alanine *	5.13 ± 0.22	3.55 ± 0.03	2.56 ± 0.04
Arginine *	4.48 ± 0.16	3.05 ± 0.01	2.10 ± 0.05
Aspartic acid *	6.10 ± 0.26	4.32 ± 0.05	3.13 ± 0.07
Glutamic acid *	9.00 ± 0.41	6.22 ± 0.13	4.49 ± 0.13
Glycine	3.21 ± 0.13	2.22 ± 0.02	1.60 ± 0.04
Proline	2.38 ± 0.10	1.70 ± 0.00	1.23 ± 0.01
cysteine	0.26 ± 0.01	0.14 ± 0.01	0.09 ± 0.01
Tyrosine	2.69 ± 0.11	1.72 ± 0.03	1.13 ± 0.04
Serine	3.20 ± 0.14	2.26 ± 0.04	1.66 ± 0.04
SRC	49.94 ± 0.17	47.73 ± 0.05	46.22 ± 0.26

SRC: ratio coefficient score of amino acids. The * represents the five dominant amino acids in *A. platensis*.

**Table 3:** Monosaccharide composition (% total monosaccharides) of *A. platensis* during culture time.

	Day 0	Day 14	Day 26
Rha	3.19 ± 0.54	1.54 ± 0.13	1.19 ± 0.09
GlcN	6.46 ± 0.34	1.65 ± 0.06	1.32 ± 0.04
Gal	11.25 ± 1.34	3.02 ± 0.24	2.17 ± 0.25
Glc	77.81 ± 0.84	92.31 ± 1.88	93.75 ± 0.96
Xyl	0.00 ± 0.00	1.16 ± 0.77	1.20 ± 0.84
GlcA	1.28 ± 1.11	0.32 ± 0.28	0.37 ± 0.32

Rha: rhamnose; GlcN: glucosamine hydrochloride; Gal: galactose; Glc: glucose; Xyl: xylose; GlcA: glucuronic acid.

**Table 4:** The content and productivity of proteins, carbohydrates, lipids, and phycobiliprotein in *A*. *platensis*.

	Content (% DW)	Volumetric Productivity (mg L^−1^ d^−1^)	Area Productivity (g m^−2^ d^−1^)	Annual Productivity (ton ha^−1^ yr^−1^)
Biomass	/	24.57 ± 1.33	3.96 ± 0.21	14.44 ± 0.78
Carbohydrates	44.78 ± 1.60	17.92 ± 0.12	2.88 ± 0.02	10.53 ± 0.07
Proteins	39.88 ± 0.72	3.75 ± 0.13	0.60 ± 0.02	2.21 ± 0.07
Lipids	3.96 ± 0.18	0.30 ± 0.01	0.05 ± 0.00	0.18 ± 0.01
Phycobiliproteins	8.14 ± 0.144	0.53 ± 0.03	0.09 ± 0.00	0.31 ± 0.02

The slash (/) represents the content can not be detected.

## Data Availability

Not applicable.

## Supplementary Materials

The following are available online at https://www.mdpi.com/article/10.3390/foods10122974/s1, Figure S1: Temperature and light intensity as a function of culture time.
