# Supplementary figures and images for: Growth and Biochemical Composition Characteristics of Arthrospira platensis Induced by Simultaneous Nitrogen Deficiency and Seawater-Supplemented Medium in an Outdoor Raceway Pond in Winter
Source: Foods. 2021 Dec 3;10(12):2974. doi: 10.3390/foods10122974 (PMC8701333; doi:10.3390/foods10122974)

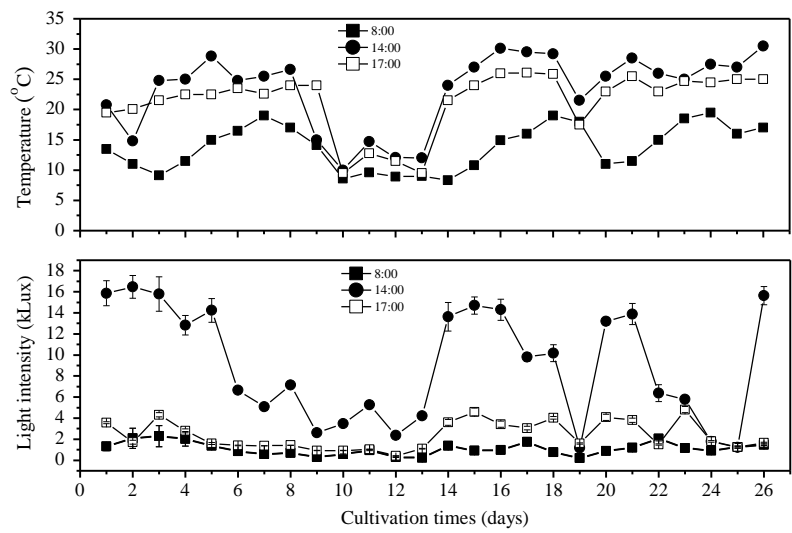

**Figure S1.** Temperature and light intensity as a function of culture time.

Supplement: Supplementary file 1 [file foods-10-02974-s001.zip › foods-1454674-supplementary.pdf]
